# A Case of Acute Amenorrhea, Pneumonia, Enlargement of the Liver

**Published:** 1847-06

**Authors:** John T. Marable

**Affiliations:** Montgomery County, Tenn.


Art. III.—*A Case of Acute Amenorrhea—Pneumonia—Enlargement of the Liver.* By John T. Marable, M.D., of Montgomery County, Tenn.

Miss P., 21 years of age, of a stout and plethoric habit, was attacked February 24th. Called to see her the same day at six o'clock in the evening, I found her laboring under the following symptoms: Weight and pain in the head and back, acute and constant pain in the region of the uterus, which was considerably aggravated by pressure over that region, short breathings, hot skin, full, hard and rapid pulse, at times delirium; cessation of the menstrual secretion.

She was bled until there was a decided impression made on the system; the blood presented a buff coat when coagulated. A cathartic composed of calomel and colocynth was given, and in about an hour and a half was followed by a pill composed of opium and tartarized antimony. In one hour from this time she became rational. She then informed me that she had been washing in cold water the day before, a thing she seldom did while menstruating; the menses ceased in about nine hours after, and the above symptoms came on. I ordered her to use the hip bath at 96°, and apply a sinapism over the region of the uterus. It is proper to remark that up to the time of her exposure the catamenia had been regular.

25th. Medicine operated well; patient slept about four hours during the night; no pain in the head; a slight soreness in the back, and somewhat tender in the region of the uterus. I ordered her to take another cathartic composed of calomel and ipecac. in the evening, to reapply the sinapism to the region of the uterus, and use the hip bath at 96°.

26th. Medicine operated well; no pain in the head or back; did not complain from pressure over the region of the uterus; menstrual secretion returned; slept well, and felt like eating. Hip bath to be used at night.

The next morning I was called to see her, the weather having suddenly grown very cold, and found her presenting all the characteristics of pneumonia. About one o'clock in the night she was taken with a chill, headache, frequent pulse, stitch in the side, some cough, and a sense of tightness and oppression about the chest. Auscultation discovered crepitant rhonchus in the left lung; percussion revealed but little if any alteration from the healthy state. Her menstruation still continues. She was bled until she complained of being faint; a cathartic composed of calomel and opium was given her; a solution of tartarized antimony to be taken every hour through the day and night; sinapism over the region of the pain.

37th. Medicine operated well; some pain in the head and chest; cough more than yesterday; sputa of a transparent color. Pill of calomel and tartarized antimony; solution of tart. antim. of greater strength to be taken as before.

28th. Medicine operated well; patient slept about four hours during the night; some little pain in the head, none in the back; sputa of a yellow brickdust color; some pain in the side; considerable cough; dullness on percussion, bronchial respiration, resonant voice. The menstrual discharge still continues. Solution of tart. antimony continued in increased strength; one grain doses of calomel to be taken every hour until her gums are slightly touched.

March 1st. Patient slept well through the night; no operation from her bowels; left side a little tender; no pain in the head or back; menstrual secretion continues; physical signs about the same as yesterday; cough not so severe; expectoration of a lighter color. Tart. antimony continued; one grain doses of calomel to be given every three hours during the day, provided her gums do not become tender; bowels to be opened with senna and manna.

2d. Patient slept well; bowels acted upon; no pain in the head or back; coughed but little during the night; sputa considerably lighter colored than yesterday; gums a little tender; menstrual secretion declining. Solution of tart. antimony to be taken every two hours.

3d. Patient rested well nearly all night; no pain in the head or back; no tenderness in the side; coughed very little; sputa nearly natural; menstrual discharge subsided.

4th. Patient better in every respect. Bowels to be kept open; solution of tart. antim. to be taken three times a day.

6th. Patient decidedly better. Bowels to be kept open; solution of tart. antimony to be taken three times a day for three or four days.

On the 25th of the month, as I was passing her house, she called me in and informed me that her stomach had been swelling for fifteen or twenty days. It had become difficult to wear her accustomed clothing. On examination, I found that her liver had from some cause become considerably enlarged. I put her upon protoiodide of mercury, under which she has been improving rapidly. I entertain no doubt, from present appearances, of her ultimate perfect recovery.

May 1, 1847.

